# Topographical distribution of anopheline mosquitoes in an area under elimination programme in the south of Iran

**DOI:** 10.1186/s12936-015-0771-7

**Published:** 2015-07-07

**Authors:** Moussa Soleimani-Ahmadi, Hassan Vatandoost, Mehdi Zare, Habibolla Turki, Ali Alizadeh

**Affiliations:** Social Determinants in Health Promotion Research Center, Hormozgan University of Medical Sciences, Bandar Abbas, Iran; Department of Medical Entomology and Vector Control, School of Public Health, Hormozgan University of Medical Sciences, P.O. Box: 79145–3838, Bandar Abbas, Iran; Department of Medical Entomology and Vector Control, School of Public Health and National Institute of Health Research, Tehran University of Medical Sciences, Tehran, Iran; Department of Occupational Health Engineering, School of Public Health, Hormozgan University of Medical Sciences, Bandar Abbas, Iran; Infectious and Tropical Diseases Research Center, Hormozgan University of Medical Sciences, Bandar Abbas, Iran

**Keywords:** Anopheles, Malaria, Topography, Minab, Iran

## Abstract

**Background:**

Malaria is a major vector-borne disease in tropical and sub-tropical countries caused by Plasmodium infection. It is one the most important health problem in south and southeast of Iran. Since Iran has recently launched to the elimination phase of malaria and vector control is one of the main strategies for elimination, this study was conducted to determine the topographical distribution of malaria vectors in Minab County, one of the important malaria endemic areas in south of Iran.

**Methods:**

In this cross-sectional study, six villages in three topographically different sites namely coastal plain, foothill and mountainous areas were selected by simple random sampling. The anopheline larvae were collected using the standard dipping method. The specimens were identified using a morphology based-key. Statistical analyses were performed using SPSS ver.16 software.

**Results:**

In total, 3,841 anopheles larvae were collected from 24 larval habitats. They consisted of ten species: *Anopheles moghulensis* (25.23%), *Anopheles stephensi* (24.47%), *Anopheles dthali* (19.14%), *Anopheles culicifacies* (9.63%), *Anopheles fluviatilis* (7.52%), *Anopheles superpictus* (5.62%), *Anopheles turkhudi* (5.55%), *Anopheles pulcherrimus* (1.93%), *Anopheles multicolor* (0.47%), and *Anopheles apoci* (0.44%). Most species were distributed in different topographies and only *An. Stephensi* and *An. culicifacies*, the main malaria vectors in Iran, were significantly associated with the altitude of studied areas. *An. moghulensis*, *An. stephensi* and *An. dthali* were the most widespread species and were, respectively predominant in Coastal plain, foothill and mountainous areas.

**Conclusion:**

Results of this study have revealed that there are many malaria vectors that are distributed in Minab County and some of them are expected to be predominant in areas with special topographic characteristics. This finding can provide a basis for effective planning and implementation of evidence-based malaria vector intervention strategies towards vector control, which may help in malaria elimination in the study area.

## Background

**Figure 1:**
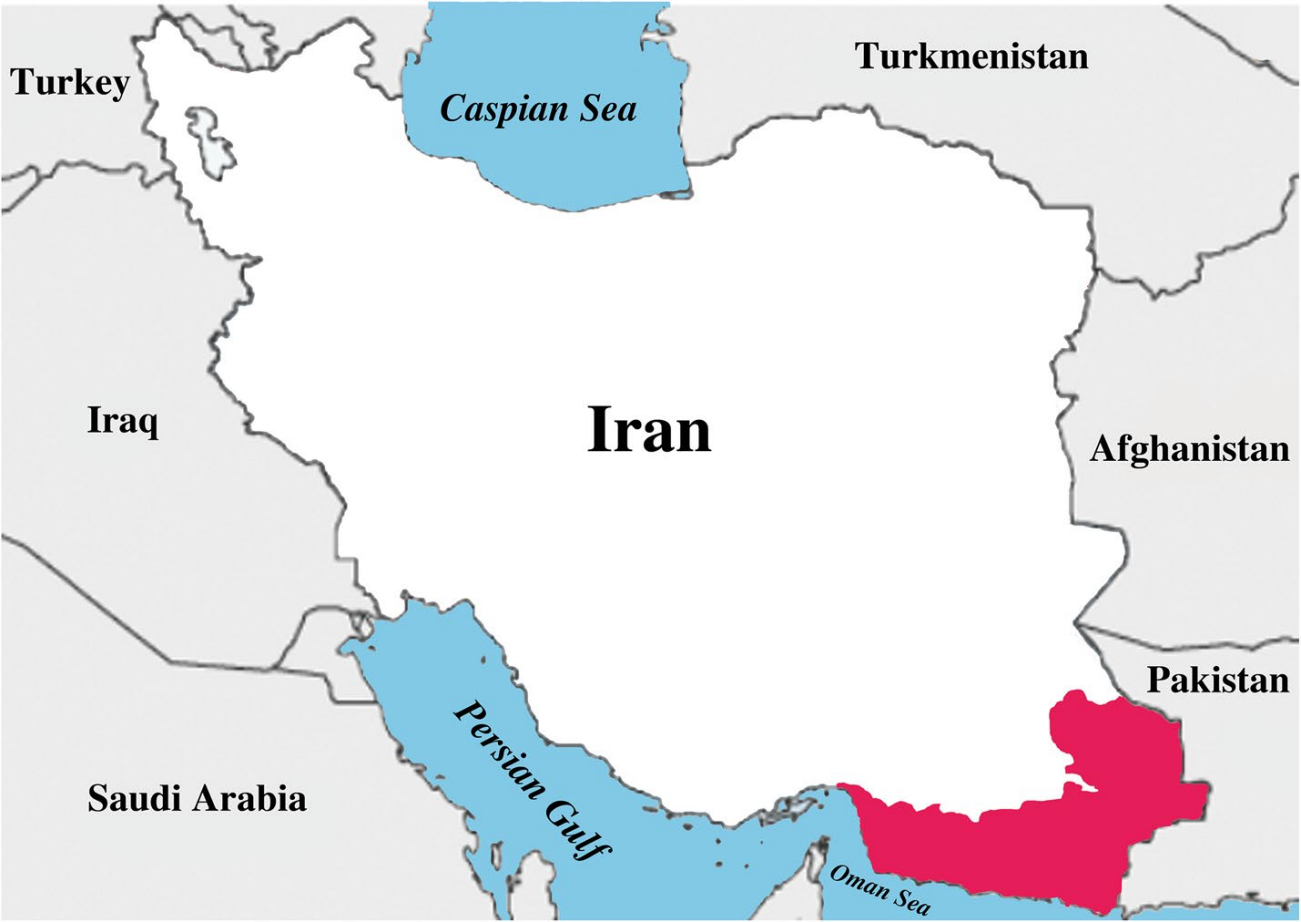
Map of Iran, *highlighting* the location of the malaria endemic areas.

Malaria is one of the most serious mosquito-borne diseases in the world, especially in the tropical and subtropical regions [1]. It is one of the foremost public health concerns in Iran and more than 90% of malaria cases are reported from the southern and south-eastern areas of the country in near border with Pakistan and Afghanistan, with two seasonal peaks mainly in spring and autumn [2] (Figure 1). According to the report of Iranian Ministry of Health and Medical Education, the annual number of malaria cases have been reduced from 12,294 in 2000 to 787 in 2012, indicating the sharp decline of disease, which has led World Health Organization to categorize Iran in the elimination phase [1, 3]. The national malaria strategic plan has recently set goals to combat malaria by taking strategies targeting vector control through indoor residual spraying (IRS), distribution of long-lasting insecticidal nets (LLINs) and application of larvicides. In this regard, Iran is aiming to eliminate *Plasmodium falciparum* by 2015 and to become malaria-free by 2025 [3, 4].

Vector control is the main way to reduce malaria transmission at the community level and in many parts of the world it is considered as the most effective measure for eradicating malaria. It has been reported to be the only measure that can reduce malaria transmission from very high levels to close to zero [1, 5]. A full understanding of environmental parameters which affect development and survival rate of the malaria vectors can be considered as a key factor in malaria control programmes. Many of these factors which are important to mosquito development and survival, such as meteorological conditions, climate, water body parameters and land use may be related to topography [6]. In general, the relationship between topography of the lands and anopheles species composition in a small geographical region is unknown. Moreover, global climatic change and environmental determinants that may be associated with human activity and alternation in land use may change anopheline species composition [7]. Therefore, it is essential to understand the distribution of anopheline mosquitoes in different topographic areas before the implementation of any vector control programme [8].

Recent studies on anopheline mosquitoes in Iran have reported the presence of 31 anopheles species including genotypes and sibling species, eight of them involved in malaria transmission [9, 10]. There are six malaria vectors in the south of Iran including *Anopheles culicifacies* s.l., *Anopheles dthali*, *Anophelesfluviatilis* s.l., *Anopheles stephensi*, *Anopheles superpictus* and *Anopheles pulcherrimus*, where *An. stephensi* and *An.culicifacies* s.l. are the principal vectors of malaria [9, 11]. Minab is a malaria endemic focus in the south of Iran and local transmission occurs in this county. Vector control strategies including IRS, LLINs and larviciding are implemented as part of an integrated approach to malaria elimination in this area. In spite of these intervention measures, malaria continues to persist in the county. Vector control is an important approach in the global malaria control programme. However, the success of this approach is dependent on the accurate and up-to-date information on the bionomics of the species involved in malaria transmission. Moreover, to support the vector control measures, information on speciation and distribution of the malaria vectors is important to ascertain what type of elimination measures are most appropriate and for which area [12].

Knowledge of the fauna and distribution of malaria vectors in different topographic areas is of particular importance for monitoring and establishment of effective control measures for malaria elimination. Therefore, this entomological study was carried out in Minab County with the aim to provide a good knowledge on the distribution of anopheline species in different topographic areas in this County. The results of this study will provide information that would help in planning and implementing an effective programme for vectors control during elimination phase by the National Malaria Control Programme.

## Methods

### Study area

The study was carried out in Minab County in the Hormozgan province, south of Iran. The county has an area of 5,393 km^2^ and is located between latitudes 26°28′–27°29′N and longitudes 56°46′–57°53′E with an approximately 320,000 population in 2013. The Minab County has a warm and humid climate with mean annual temperature of 26.5°C ranging from 19.9 to 33.1°C. The rainfall occurs through the November–March with an annual average of 40.6 mm. The averages of minimum and maximum relative humidity are 31% in May and 60% in November, respectively. Minab is an agricultural region irrigated by rivers, wells and cement pools, which are the main breeding sites for anopheline mosquitoes. In this county nearly all of houses are made of cement and blocks and have electricity and water supply. Agriculture, livestock herding and fishing are the main occupations in the study area. Minab is one of the most important malaria endemic areas in the south of Iran and malaria cases are reported in this area year-round with peaks after the two annual rainy seasons (April–June and October–December) [3].

### Study design

**Figure 2:**
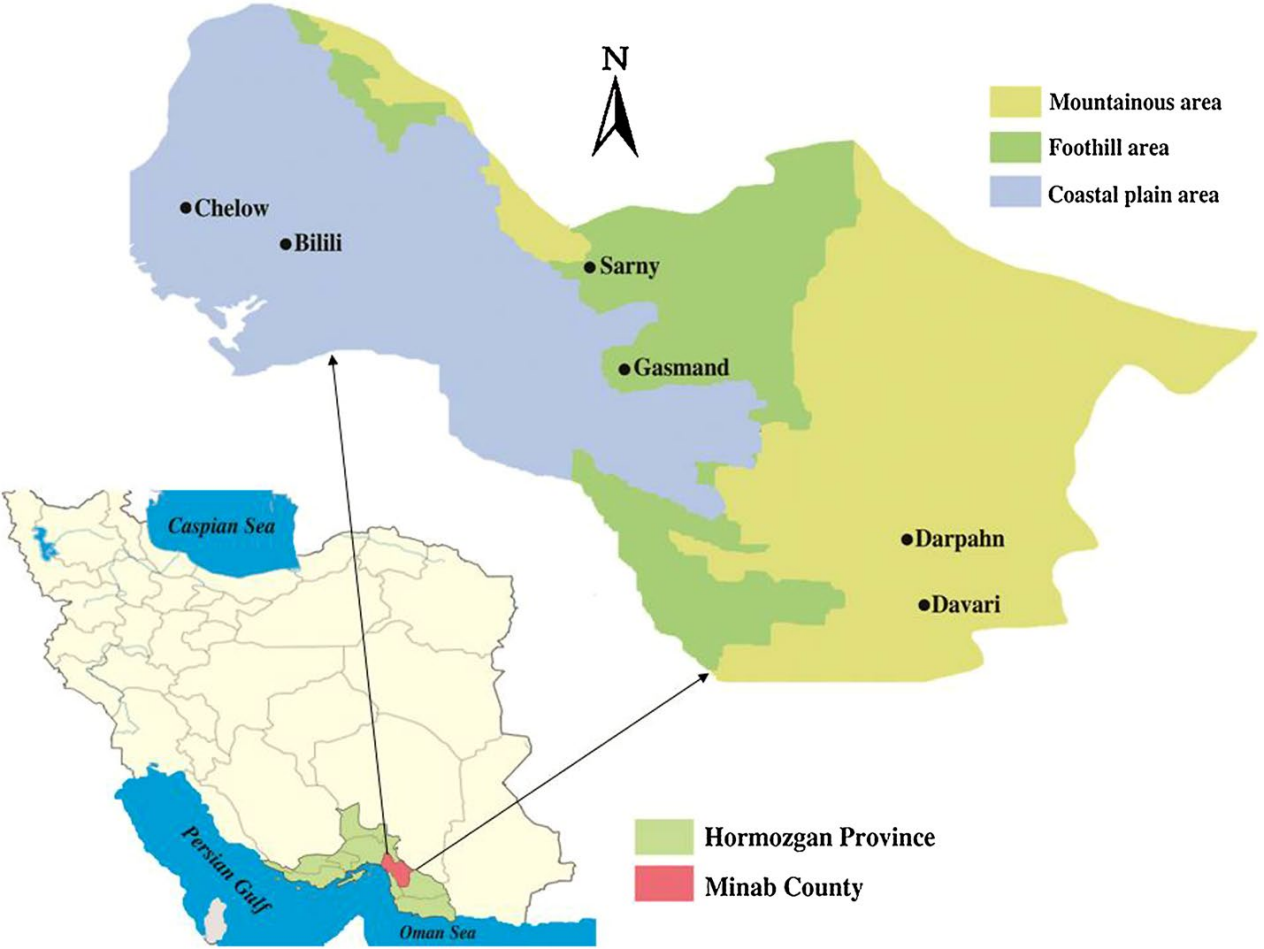
Map showing the provinces of Iran, *highlighting* the location of Hormozgan province and study villages in Minab County.

In this study six villages in different topographical regions in Minab County were selected by simple random sampling based on reported malaria cases during the past years, human population densities and suitability of the places for mosquito collection. Anopheline larvae were collected from selected villages including: Chalow (27°11′N, 56°58′E, 9 m) and Balili (26°04′N, 57°01′E, 15 m) in a coastal plain area, Sarney (27°05′N, 57°21′E, 240 m) and Gasmand (26°46′N, 57°13′E, 155 m) in a foothill area and Darpahn (26°35′N, 57°39′E, 465 m) and Davari (26°35′N, 57°36′E, 680 m) in a mountainous area (Figure 2).

### Larval sampling and identification

Anopheline specimen collection was performed at different times during the mosquito breeding season from September 2012 to August 2013. In each village all larval habitats present in and within a 500-m radius of the village were sampled for anopheline larvae using a standard 350 ml capacity mosquito dipper or a white plastic pan with the same capacity according to WHO procedures [13]. When mosquito larvae were present, 10–30 dips were taken depending on the size of each larval habitat at intervals along the edge. In small breeding places where dippers were not effective, larval collection was performed using plastic pipettes. Samplings were always done by the same individual in the morning (08:00– 12:00 h) or afternoon (14:00–17:00 h) for about 30 min at each larval habitat. All third and fourth instars of anopheline larvae were passed though a 100 mesh sieve and preserved in lacto-phenol. In the laboratory, each larva was individually mounted in Berlese’s medium on a microscope slide and identified to species by morphological characters [14].

### Statistical analysis

The data were analysed using SPSS Ver. 16. Chi squared analyses were used to test the relationship between mosquito density and topographic type. Larval densities were calculated as number of larvae per 10 dips.

## Results

### Anopheles species composition and abundance

**Figure 3:**
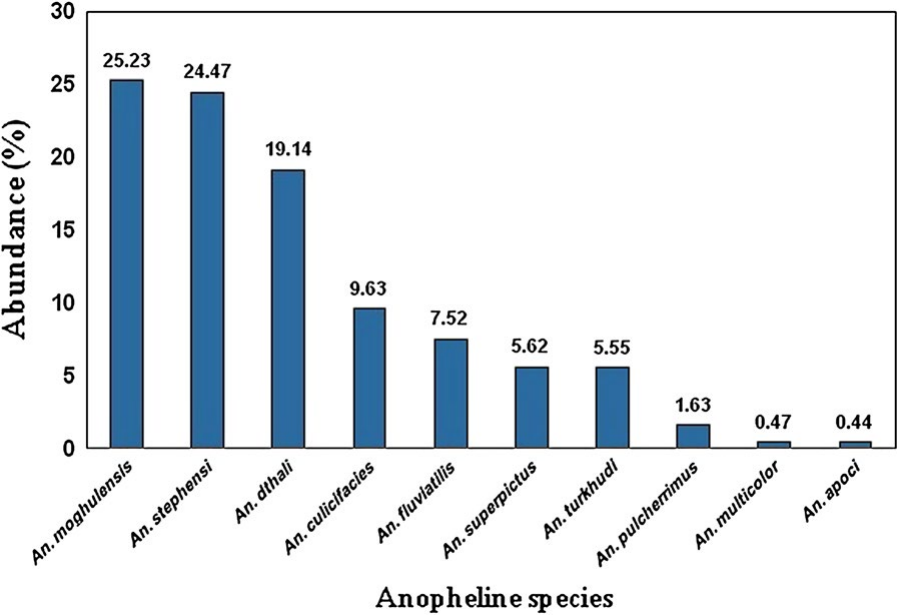
Abundance of the Anopheline species in Minab County, South of Iran.

During this study, a total of 3,841 anopheline larvae, representing ten species were collected and identified. As shown in Figure 3, the most abundant species were *Anopheles moghulensis* (25.23%), *An. stephensi* (24.47%), and *An. dthali* (19.14%), which together accounted for 68.84% of the total anopheline collected. The least collected species were *An. culicifacies* s.l. (9.63%), *An. fluviatilis* s.l. (7.52%), *An. superpictus* (5.62%), *Anopheles turkhudi* (5.55%), *An. pulcherrimus* (1.63%), *Anopheles multicolor* (0.47%), and *Anopheles apoci* (0.44%).

### Topographical distribution and abundance of anopheles species

**Figure 4:**
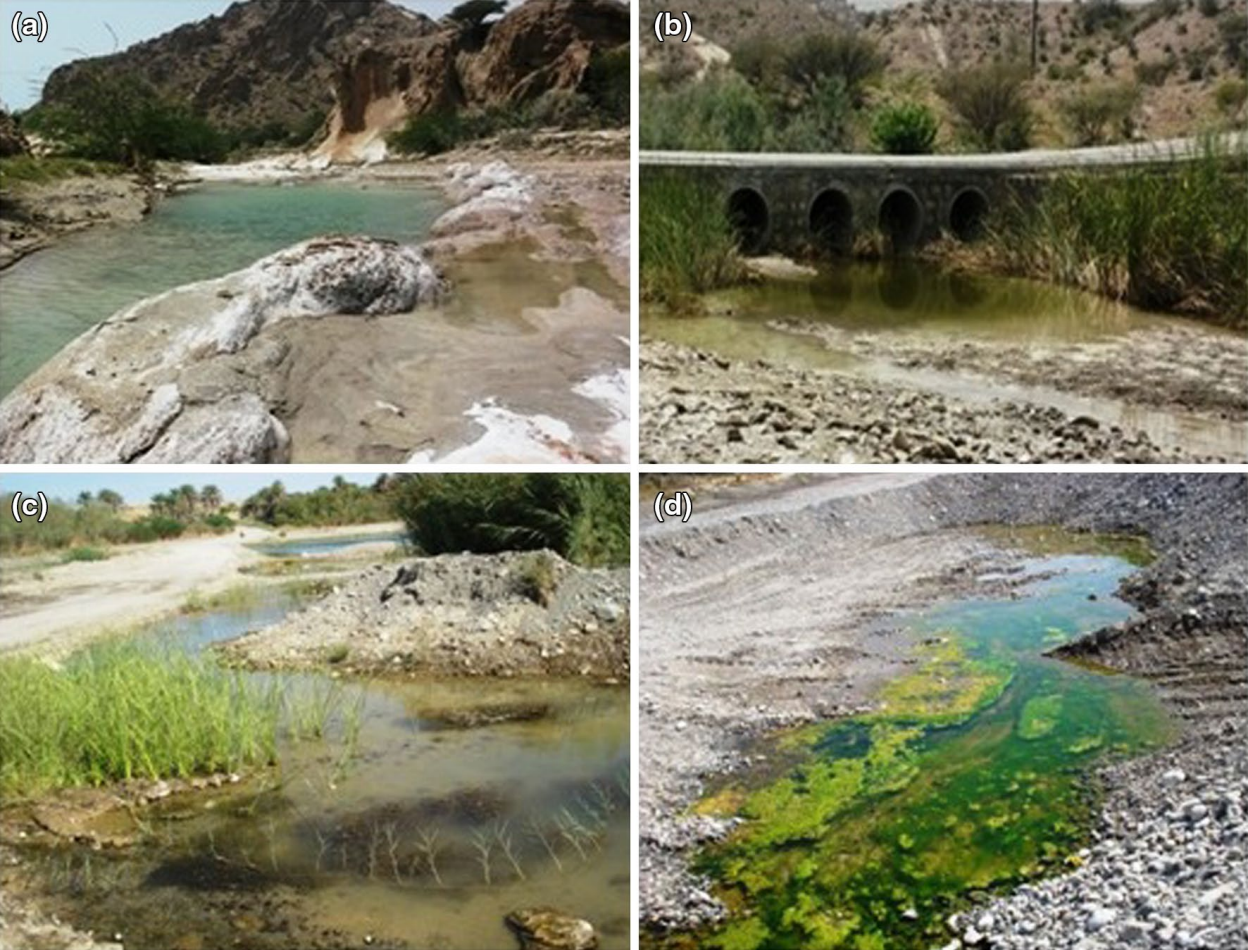
Typical potential anopheline larval habitats in different topographical areas in Minab County, south of Iran. Mountainous (**a**), foothill (**b**) and coastal plain (**c**, **d**).

In present study, Anopheline larvae were collected from natural habitats in different topographical areas (Figure 4). The high numbers of anopheles species were collected in foothill regions (Table 1). This species included *An. dthali* (23.67%) and *An. stephensi* (22.26%), which were the most frequently collected species, followed by *An. moghulensis* (13.43%), *An. fluviatilis* (12.78%), *An. turkhudi* (10.02%), *An. culicifacies* (8.81%), *An. pulcherrimus* (4.98%), *An. superpictus* (3.23%) and *An. multicolor* (0.8%).

**Table 1:** Abundance of the anopheline mosquitoes in different topographical areas of Minab County, south of Iran

Species	Topographic areas			
	Coastal plain no. (%)	Foothill no. (%)	Mountainous no. (%)	Total no. (%)
*An. moghulensis*	407 (32.46)	200 (13.45)	362 (32.90)	969 (25.23)
*An. stephensi*	455 (36.28)	331 (22.26)	154 (14)	940 (24.47)
*An. dthali*	181 (14.43)	352 (23.67)	202 (18.36)	735 (19.14)
*An. culicifacies*	0	131 (8.81)	239 (21.73)	370 (9.63)
*An. fluviatilis*	77 (6.14)	190 (12.78)	22 (2.00)	289 (7.52)
*An. superpictus*	123 (9.81)	48 (3.23)	45 (4.1)	216 (5.62)
*An. turkhudi*	11 (0.88)	149 (10.02)	53 (4.84)	213 (5.55)
*An. pulcherrimus*	0	74 (4.98)	0	74 (1.93)
*An. multicolor*	0	12 (0.8)	6 (0.55)	18 (0.47)
*An. apoci*	0	0	17 (1.54)	17 (0.44)
Total	1,254 (100)	1,487 (100)	1,100 (100)	3,841 (100)

Results of anopheles species composition and their abundance in coastal plain area showed that *An. stephensi* (36.28%) was predominant and widely distributed species. The other three common species were *An. moghulensis* (32.46%), *An. dthali* (14.43%), and *An. superpictus* (9.81%). *Anopheles fluviatilis* and *An. turkhudi* were the least abundant species which contributed 6.14 and 0.88% of the total collection, respectively (Table 1). Moreover, in this study 1,100 anopheline larvae, belonging to nine species, including *An. moghulensis*, *An. culicifacies*, *An. dthali*, *An. stephensi*, *An. turkhudi*, *An. superpictus*, *An. fluviatilis*, *An. apoci* and *An. multicolor* were collected in the mountainous area. The abundance of the anopheline specimens which collected in mountainous area are shown in Table 1.

Mean density of anopheline larvae in the three topographical areas are shown in Table 2. The least and the most anopheline larval mean density were 3.43 ± 0.37 and 4.64 ± 0.38 larvae/10 dips in mountainous and foothill areas, respectively. Although the mean larval density of each anopheles species was variable, there was no significant relationship between topographic type and anopheles specimen’s density in the study areas (*P* = 0.897).

*Anopheles stephensi* was found predominantly in coastal plain regions and it was also distributed with varying densities in foothill and mountainous areas (Table 2). Distribution and frequency of *An. stephensi* had a significant relationship with topographic types in the study areas (*P* = 0.023). *Anopheles dthali* was found prevalently with varying densities throughout the study areas. This species was also more common in foothill regions and its larval densities were generally high in the moderately high altitude localities (Table 2). No significant relationship was found between mean larval density of *An. dthali* and topographic type in the study areas (*P* = 0.614). *Anopheles culicifacies* s.l. was collected more frequently from mountainous than foothill regions and it was absent in coastal plain localities (Table 2). Increasing altitude was significant factor in the abundance and distribution of *An. culicifacies* in the studied areas (P = 0.046).

**Table 2:** Mean density of anopheline larvae in Minab County, south of Iran (larvae/10 dips)

Species	Topographic areas				*P*
	Coastal plain	Foothill	Mountainous	Total	
*An. moghulensis*	10.17 ± 1.13	5.14 ± 0.82	9.05 ± 1.15	24.21 ± 3.12	0.521
*An. stephensi*	11.37 ± 1.62	8.27 ± 0.73	3.85 ± 0.56	23.50 ± 2.96	0.023
*An. dthali*	4.52 ± 0.64	8.80 ± 1.05	5.05 ± 0.46	18.37 ± 2.86	0.614
*An. culicifacies*	0	3.27 ± 0.58	5.97 ± 0.86	9.25 ± 1.35	0.046
*An. fluviatilis*	1.92 ± 0.21	4.75 ± 0.63	0.56 ± 0.02	7.22 ± 1.92	0.641
*An. superpictus*	3.07 ± 0.46	1.23 ± 0.34	1.12 ± 0.27	5.40 ± 1.32	0.825
*An. turkhudi*	0.27 ± 0.02	3.17 ± 0.68	1.33 ± 0.42	5.32 ± 0.61	0.764
*An. pulcherrimus*	0	1.85 ± 0.21	0	1.85 ± 0.21	–
*An. multicolor*	0	0.30 ± 0.02	0.15 ± 0.01	0.45 ± 0.03	0.986
*An. apoci*	0	0	0.42 ± 0.05	0.42 ± 0.05	–
All species	3.92 ± 0.38	4.64 ± 0.51	3.43 ± 0.37	12.10 ± 1.42	0.897

Mean density of anopheline larvae is expressed as mean ± SE.

*Anopheles fluviatilis* s.l. was collected from various localities in studied areas but it was more prevalent in foothill regions (Table 2). Statistical analysis indicated that there was no significant relationship between abundance of *An. fluviatilis* and altitude of studied areas (*P* = 0.641). *Anopheles superpictus* was distributed in the study area but it was more prevalent in the coastal plain locality (Table 2). There was no significant difference between mean larval density of *An. superpictus* and topographic regions (*P* = 0.825). *Anopheles pulcherrimus* was found in low density with a distribution restricted to the foothill regions.

*Anopheles moghulensis*, a non-vector species, was the most abundant species and distributed in various topographic regions (Table 2). Chi squared analyses showed that there was no significant relationship between abundance of *An. moghulensis* and topographic type in the study areas (*P* = 0.521). *Anopheles turkhudi*, another non-vector species, was captured from all of studied regions but it was more prevalent in foothill regions. However, there was no significant relationship between altitude of locations and distribution of *An. turkhudi* in the studied areas (P = 0.764). *Anopheles multicolor*, the third found non-vector species, was associated with low larval density in the foothill and mountainous regions (Table 2). There was no significant association between mean larval densities of *An. multicolor* and topographic region (*P* = 0.986). *Anopheles apoci*, the fourth non-vector species, was less common with a distribution restricted to the mountainous regions (Table 2).

## Discussion

In this study, topographical distribution of anopheles species in Minab County was determined. Such studies are important for the implementation of targeted vector control interventions, especially in this county where several large campaigns such as massive distribution of long-lasting insecticidal nets and indoor residual spraying are currently supported by national malaria programme and Global Fund.

During this study, ten anopheline species were identified in the Minab County, which included six out of the eight known malaria vector species in Iran, namely *An. stephensi*, *An. dthali*, *An. culicifacies*, *An. fluviatilis*, *An. superpictus* and *An. pulcherrimus. Anopheles stephensi* is considered as the primary vector and other species, *An. dthali*, *An. culicifacies*, *An. fluviatilis*, *An. superpictus*, and *An. pulcherrimus*, play the main role as secondary vectors in the south and south-east of the country [9, 11]. The high diversity and abundance of malaria vectors in the study area is probably due to the special geographical position of Minab County which is located at the confluence of three Zoogeographical regions including Palaearctic, Afrotropical and Oriental which provides suitable conditions for proliferation of different anopheline mosquitoes.

During the study, anopheline species were abundantly collected from foothill area. In this area mosquito breeding places were mainly rivers that in most of the times have low levels of water which creates favourite conditions for mosquitoes breeding. Similar results have been reported from Bashagard, the neighbouring county of the study area [15].

The results showed that three most commonly collected species, including *An. moghulensis*, *An. stephensi* and *An*. *dthali* had wide distribution and collected from all of topographic regions. *Anopheles stephens* was prevalent in coastal plain and with varying densities throughout the study areas. The same abundance and distribution were reported in previous studies in the malarious area of southern Iran up to altitude of 900 m [9]. Although there is no evidence of hibernation in this species and probably it breeds throughout the year, its density is decreased in cold weather. One of the main reasons for prevalence of *An. stephensi* in Minab county may be that its life is adaptable to a wide range of larval habitats, and surface water bodies especially Minab river provide favourite situations for its breeding. Moreover, high prevalence of this species in coastal plain may be due to its tolerance to humid environment. This finding agree with results from Arabian peninsula and South Asian region, including Indian subcontinent and Pakistan, which has the same climate pattern as that of the south of Iran [16]. *Anophelesstephensi* has been confirmed as the main malaria vector in coastal area of the south of Iran and infection rate of this species with *Plasmodium vivax* and mixed infection with *P. vivax* and *P. falciparum* in the study area has been reported to be 0.97 and 0.32%, respectively [17].

In current study, *An*. *dthali* was abundantly collected from foothill regions. This agrees with a previous study by Yaghoobi-Ershadi et al. [9, 18] carried out in the study area and conforms to the well distribution of *An*. *dthali* in southern parts of the Zagros chain and coastal area of the Persian Gulf up to 1,410 m. This species has been introduced as a secondary vector in southern Iran and in northern regions of Somalia and Saudi Arabia [11]. Sporozoite rate for this anopheles in southern Iran has been reported to be 0.67–2.08% [9]. Therefore, despite its contribution to malaria transmission in the studied area, it is unlikely that this species could play a significant role in malaria transmission in the absence of the major vectors in the study area.

According to results, among the secondary vectors, *An. culicifacies* can be a potential vector since its role in malaria transmission has been reported from Sistan va Baluchestan province in the southeast of the country bordering Afghanistan and Pakistan. This species is largely responsible for an epidemy of malaria in Sistan and Baluchestan province [11]. Sporozoite rate for this species is reported 1–4.7% in the south and southeast of Iran [9]. *Anopheles culicifacies* has also known as a malaria vector with wide distribution in the Asia and Indian subcontinent [19]. Compared to the results of other studies conducted in Hormozgan province, the relative abundance of *An. culicifacies* in the mountainous areas in the present study is high [20]. Similar abundance has been reported for this species from Iranshahr, the neighboring district to the study area [21].

Our study showed that the *An. fluviatilis* was common in Minab County and it was found predominantly on the foothill regions. Distribution of this species in foothill regions has already been reported at altitude 50–1,100 m, along the foothills of the Zagros Mountains that extend from south to southeast of Iran. This species was considered as a secondary vector in the south and southeast of Iran and its sporozoite rate is reported to be 1.4–11% [9].

*Anopheles superpictus* is known as a main malaria vector in the centre of Iran, and the secondary vector in the southern areas of the country and sporozoite rate for this species is reported to be 0.65–4.7% in the country [9]. In this study, *An. superpictus* was collected with varying densities from all of topographic regions. It is the most widespread anopheles species in Iran and presents in almost all parts of the country in altitudes 50–2,000 m across all climatic zones including arid to wet climates [22]. This species has a broad geographical distribution in Asia, Europe and northern Africa, and has been recognized as a malarial vector in these regions [23].

Findings showed that *An. pulcherrimus* is present only in foothill regions. It has been reported as a malaria vector in Ghasreghand district in the east of study area with sporozoite rate of 3.5% [24]. This anopheles has been known to be a malaria vector in Iraq and Afghanistan [25]. It has a wide distribution in western Asia, extending from Lebanon, Syria and Iraq in the west, to Iran, Afghanistan, Pakistan and India in the east. It has been also found in Turkey and Turkmanistan and Caucasus in the north and Saudi Arabia and Bahrain in the south [26]. Since this species is abundant in moderate and highly semi-arid climates [9], it seems humidity is a restricting factors for distribution of this species in the Coastal plain of the study area. *Anopheles moghulensis* was predominant across the study area. Similar results are also reported in the neighbouring areas, where it has been reported to be widely distributed [15]. This species has never been as a malaria vector in Iran because of its exophilic and zoophilic behaviour [11, 15].

*Anopheles turkhudi* was distributed in the entire study region but it was collected more often in the foothill regions. This species is not considered to be a vector of malaria in Iran and it is distributed in the southern and some central areas of the country [10]. *Anopheles multicolor* was collected in relatively few numbers in the study area. This species has been reported mainly in the east of Iran [27]. This anopheles also has not been confirmed as a malaria vector in the country. *Anopheles apoci* was captured with low frequency in the mountainous regions. It is distributed in the southern Iran and is not considered as a malaria vector in the country [10].

In the present study, although some species are found in similar areas, they show different abundance over those areas. This may be due to different topographic characteristics of areas including the altitude, but other environmental and biological factors such as temperature, larval habitat characteristics and preferred host may influence distribution and abundance of anopheline mosquitoes which have not been considered in this study. Moreover, human activity including farming and livestock rearing and climate change may have influence on the distribution of anopheles species.

## Conclusions

The current study demonstrated diversity and abundance of anopheles species in different topographic areas in study area. According to the results, some species showed different abundance and distribution over different topographic areas. This study showed that some of malaria vectors are expected to be predominant in areas with special topographic characteristics. This finding can provide a basis for appropriate intervention towards vector control, which may help in suppression of vector density, and consequently, malaria elimination in the study area. Furthermore, these findings would have impact on the effective planning and implementation of evidence-based malaria vector intervention strategies and elimination of malaria in Iran.
